# The Therapeutic Potential and Limitations of Ketones in Traumatic Brain Injury

**DOI:** 10.3389/fneur.2021.723148

**Published:** 2021-10-22

**Authors:** Savannah Anne Daines

**Affiliations:** ^1^Department of Biology, Utah State University, Logan, UT, United States; ^2^Department of Kinesiology and Health Science, Utah State University, Logan, UT, United States

**Keywords:** cerebral metabolism, glucose, ketogenic, ketone, ketosis, traumatic brain injury

## Abstract

Traumatic brain injury (TBI) represents a significant health crisis. To date, no FDA approved pharmacotherapies are available to prevent the neurological deficits caused by TBI. As an alternative to pharmacotherapy treatment of TBI, ketones could be used as a metabolically based therapeutic strategy. Ketones can help combat post-traumatic cerebral energy deficits while also reducing inflammation, oxidative stress, and neurodegeneration. Experimental models of TBI suggest that administering ketones to TBI patients may provide significant benefits to improve recovery. However, studies evaluating the effectiveness of ketones in human TBI are limited. Unanswered questions remain about age- and sex-dependent factors, the optimal timing and duration of ketone supplementation, and the optimal levels of circulating and cerebral ketones. Further research and improvements in metabolic monitoring technology are also needed to determine if ketone supplementation can improve TBI recovery outcomes in humans.

## Introduction

Traumatic brain injury (TBI) occurs when an insult to the head disrupts normal brain function. The Centers for Disease Control and Prevention reports that 2.87 million TBIs are diagnosed each year, with age adjusted TBI emergency room visits increasing 54% in recent years (1, 2). These numbers do not account for individuals who did not seek care, received outpatient or office-based care, or received care at federal facilities, such as those serving in the U.S. military or receiving care at a Veterans Affairs hospital (1). Because milder forms of TBI, including concussions, are often treated outside of emergency rooms, and because those who serve in the U.S. military are at significant risk for TBI, TBI incidence is underestimated (1).

TBIs are significant injuries that may result in long-term consequences, including neurodegeneration, cognitive dysfunction, and comorbid neuropsychiatric illnesses. It is estimated that 5.3 million individuals in the United States have a TBI-related disability, which places a sizable socioeconomic burden on patients, healthcare systems, and society (1, 3). Developing treatments that would help TBI patients recover faster and more completely would reduce healthcare costs, benefit society as a whole, and improve TBI patients' quality of life. However, TBIs are challenging to diagnose, treat, and study because TBIs present with a complex, multifaceted nature manifested through diverse clinical presentations, injury mechanisms, and factors specific to the individual (4). The path to developing better TBI treatments involves elucidating the underlying mechanisms of the brain's response to TBI in order to target specific pathophysiological responses.

## Primary and Secondary Mechanisms of Injury

**Figure 1:**
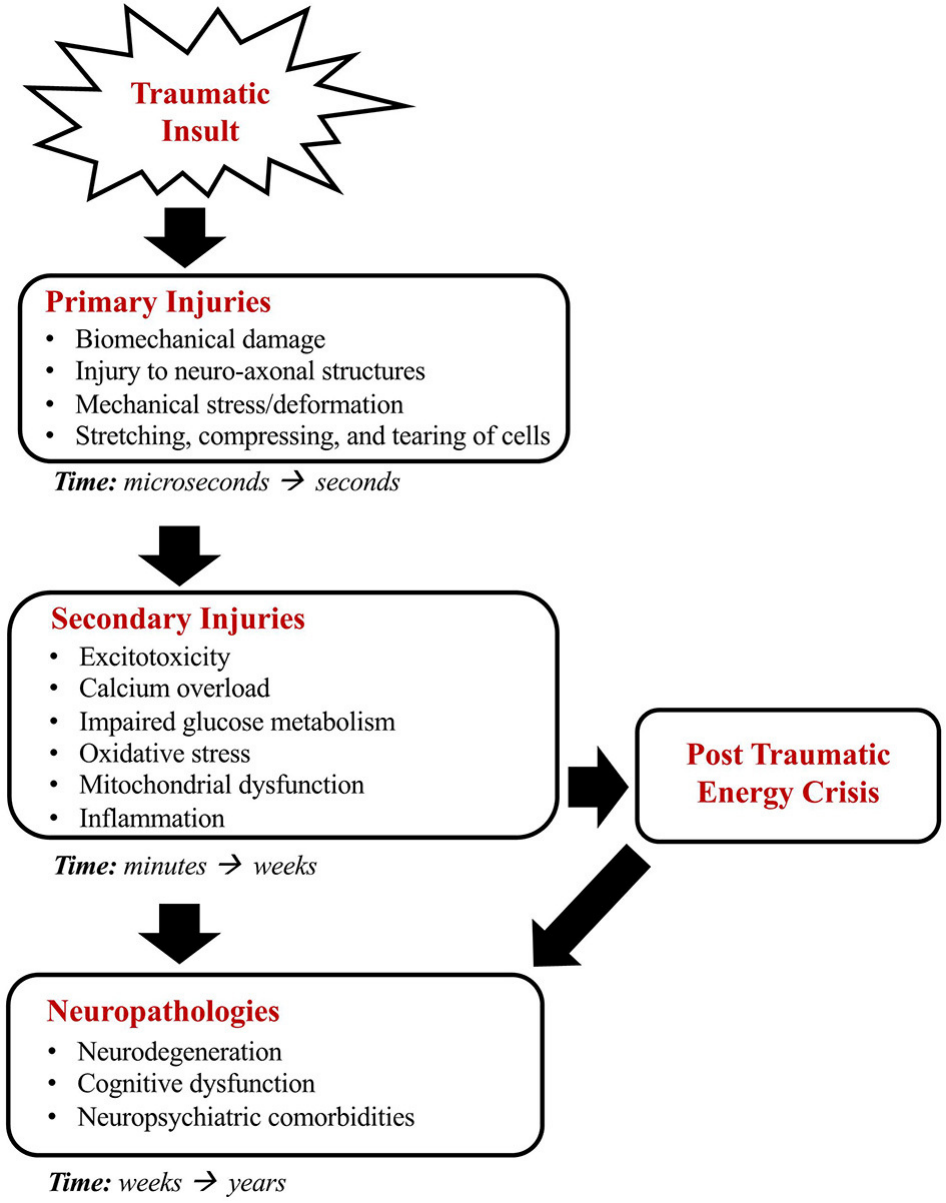
A schematic representation of injury processes following a TBI. The mechanical forces of the traumatic insult result in primary injuries characterized by deformations in brain tissue. Following the primary injury, secondary injuries ultimately result in cerebral energy deficiencies and long-term sequelae.

The consequences of TBIs can be classified as either primary or secondary injuries. Primary injuries result directly from mechanical forces, including shearing and direct impact forces, at the moment of the traumatic event. Preventative measures, such as using protective equipment, can reduce the severity of the injury. However, brain tissue deformations from primary injuries are largely irreversible. Secondary injuries occur indirectly as a response to primary injuries and are characterized by interconnected cascades of biochemical, cellular, and molecular events that cause further damage but have the potential to be minimized and reversible (5, 6). Secondary injuries include an excessive release of excitatory neurotransmitters, calcium overload, glucose dysmetabolism, free radical overproduction, mitochondrial dysfunction, and neuroinflammation (7). These secondary injury responses deplete cerebral energy stores and can result in long-term neuropathological consequences. Figure 1 schematically illustrates the time-courses and relationship of primary and secondary injuries in TBI.

### Cerebral Metabolism in Secondary Injuries

The cascade of secondary injury mechanisms begins to occur immediately following the primary injury. The force of the primary injury triggers the release of glutamate in neurons and disrupts the integrity of the plasma membrane resulting in an efflux of potassium and influx of sodium and calcium (7, 8). Sustained glutamate release in damaged neurons activates ionotropic N-methyl-D-aspartate (NMDA) and α-amino-3-hydroxyl-5-methyl-4-isoxazole-propionate (AMPA) receptors (7, 9). The opening of these channels increases the ionic flux, further disrupting the ionic equilibrium and compounds the need for ion pumps to use adenosine triphosphate (ATP) to restore ionic and cellular homeostasis (8). The increased demand for ATP initially results in compensatory accelerated glucose metabolism, a state referred to as hyper-glycolysis (8). However, these neurochemical events quickly deplete cellular energy stores by creating a mismatch between energy supply and demand (8).

A hypometabolic stage begins around 6 hours post-injury with its duration being dependent upon injury severity (10). This stage is marked by glucose dysmetabolism and mitochondrial dysfunction. Failure of autoregulation mechanisms that maintain cerebral blood flow despite changes in cerebral perfusion pressure [cerebral perfusion pressure (CPP) = mean arterial pressure (MAP) − intracranial pressure (ICP)] results in decreased cerebral blood flow (11). Decreased cerebral blood flow reduces oxygen and glucose delivery to the brain, limiting glucose metabolism (7). Mitochondrial dysfunction results from excessive intracellular calcium accumulation (7). Excessive intracellular calcium is sequestered into the mitochondria where its accumulation opens the mitochondrial permeability transition pore (MPTP) and inhibits electron transport chain complexes, leading to a cerebral energy crisis and the initiation of apoptotic and necrotic cell death (12–14).

**Figure 2:**
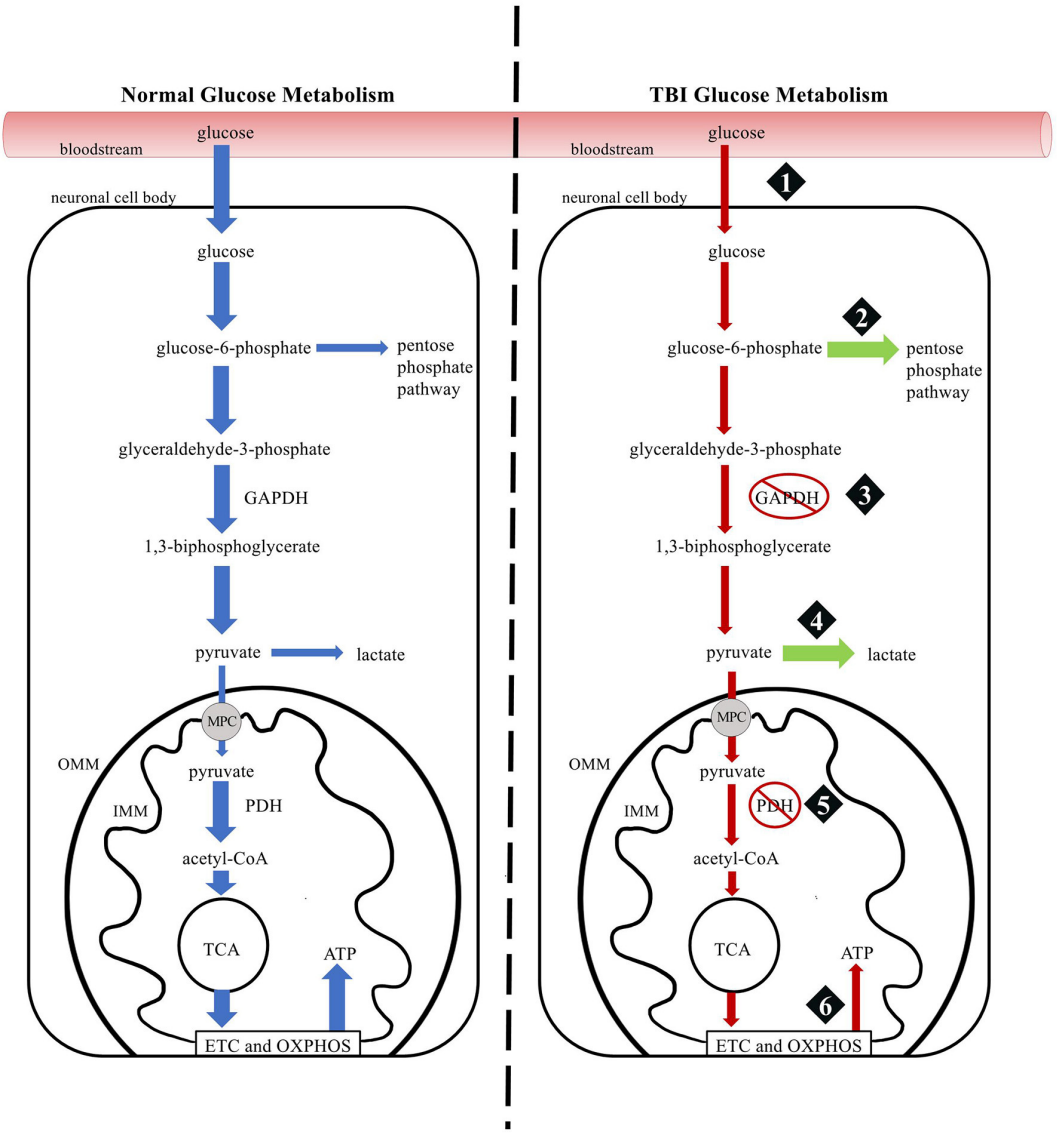
Comparison of glucose metabolism in normal and TBI physiology. (1) A TBI triggers a transient increase in glucose uptake followed by a prolonged decrease in glucose metabolism. (2) Following a TBI, a larger portion of glucose-6-phosphate is shunted to the Pentose Phosphate Pathway to generate protective and reparative molecules. (3) Glyceraldehyde-3-phosphate dehydrogenase is slowed, decreasing tricarboxylic acid cycle intermediates. (4) Lactate production increases in astrocytes so that lactate can be shuttled to energy-deficit neurons. (5) The pyruvate dehydrogenase complex is inhibited, decreasing tricarboxylic acid cycle intermediates. (6) The function of the electron transport chain and oxidative phosphorylation is impaired, resulting in less ATP production. GADPDH, glyceraldehyde-3-phosphate dehydrogenase; MPC, mitochondrial pyruvate carrier; PDH, pyruvate dehydrogenase complex; TCA, tricarboxylic acid cycle; ETC, electron transport chain; OXPHOS, oxidative phosphorylation; ATP, adenosine triphosphate; OMM, outer mitochondrial membrane; IMM, inner mitochondrial membrane.

The impairment of oxidative phosphorylation leads to the production of reactive oxygen and nitrogen species that exceed the cell's antioxidant defenses (7, 15). As reactive oxygen and nitrogen species damage intracellular structures, repair enzymes are activated that use and deplete intracellular nicotinamide adenine dinucleotide (NAD+) stores (16). The decrease in NAD+ slows the ability of glyceraldehyde-3-phosphate dehydrogenase to catalyze glyceraldehyde 3-phosphate to 1,3-biphosphoglycerate in glycolysis, which disrupts glucose metabolism (16, 17). The ability of glucose to generate ATP is further disrupted as glucose is shunted to the pentose phosphate pathway at an increased rate in response to oxidative damage, as more pyruvate is converted to lactate in astrocytes to shuttle lactate to energy-deficit neurons, and as the pyruvate dehydrogenase complex is inhibited in response to oxidative stress (18–20). Figure 2 provides a schematic comparison of normal glucose metabolism and TBI glucose metabolism.

The observed impairments at numerous points in the glycolytic pathway and oxidative metabolism of glucose suggest that a more optimal substrate could be used as fuel after a traumatic brain injury. Considering that secondary injury processes are interconnected and have downstream consequences, interventions targeting specific secondary injury mechanisms could improve TBI treatment outcomes. Because the reduction of cerebral energy has significant adverse consequences and is a strong, independent predictor of poor outcomes associated with neuropathology, cognitive deficits, and functional decline, establishing treatment methods to target cerebral energy deficits could help improve TBI recovery (21–23).

## Ketones

**Figure 3:**
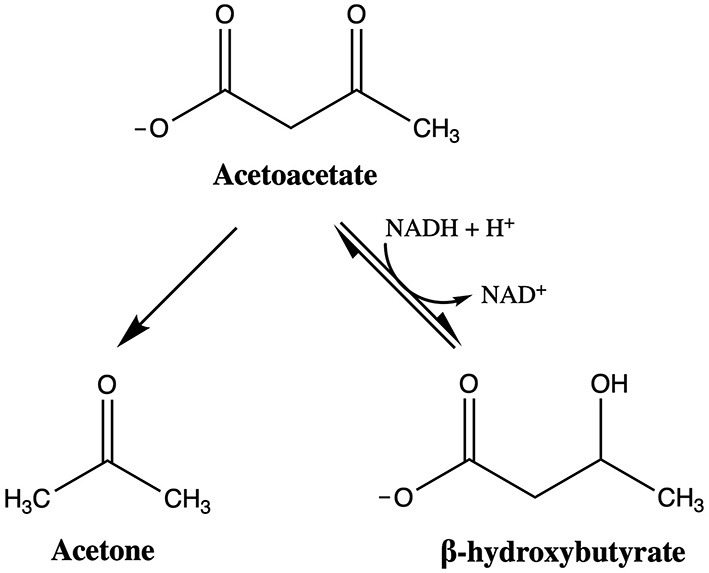
Interconversion and structure of ketone bodies. If acetoacetate is not oxidized to acetyl-CoA to enter the tricarboxylic acid (TCA) cycle, acetoacetate can convert to acetone or β-hydroxybutyrate. Acetoacetate decarboxylase catalyzes the reaction of acetoacetate to acetone, which is exhaled or excreted as waste. The interconversion of acetoacetate and β-hydroxybutyrate is catalyzed by β-hydroxybutyrate dehydrogenase, with the extent of the reaction dependent upon the cellular pool of NAD+. If the reaction favors acetoacetate, acetoacetate is oxidized to acetyl-CoA and enters the TCA cycle to generate energy.

Ketone bodies are an alternative cerebral energy substrate that can target secondary injury mechanisms at various levels. Endogenous ketone bodies are synthesized in the body *via* lipolysis of free fatty acids in hepatic mitochondria and locally within astrocytes (24, 25). There are three ketone bodies: acetoacetate, β-hydroxybutyrate, and acetone. Figure 3 depicts the interconversion and structure of ketone bodies. When in the metabolic state of ketosis, β-hydroxybutyrate constitutes the majority of ketone bodies produced and is a reliable indicator of when the body is in ketosis.

Under normal dietary conditions with available carbohydrate stores, the production of ketone bodies is low. However, if the body has low carbohydrate stores, the body enters into a state of ketosis, where the production of ketone bodies is upregulated (26). Because carbohydrate metabolism is disrupted in TBIs, if the body can switch to ketosis, ketone bodies can function as an alternative energy source to glucose.

In terms of clinical use, the benefits of endogenously produced ketone bodies are limited due to the timeframe of inducing ketosis through a ketogenic diet containing low carbohydrates and high fats in the form of medium chain triglycerides. Endogenous ketone body production through a ketogenic diet and/or fasting takes 3–5 days to reach therapeutic blood ketone body levels (27). However, exogenous ketone supplementation can achieve therapeutic levels of blood ketone levels within 30 minutes of administration (28, 29). Exogenous ketones exist as liquids or powders that contain ketone bodies or ketone body precursors, including medium chain triglycerides, ketone mineral salts, and β-hydroxybutyrate esterified to precursor molecule. These compounds are clinically useful as they increase circulating levels of β-hydroxybutyrate without significantly lowering levels of insulin or glucose (27).

Medium chain triglycerides are comprised of saturated fatty acids containing 6–10 carbon atoms that are rapidly absorbed and converted to ketone bodies in the liver (30). A 30 g dose of medium chain triglycerides can raise circulating levels of β-hydroxybutyrate to 0.5 mM (31, 32). However, the dosage is limited due to poor gastrointestinal tolerability.

Ketone salts typically comprise β-hydroxybutyrate bound to a salt such as sodium, potassium, or calcium and can be administered orally or intravenously. They are widely available as a supplement or prescription. Oral administration of ketone salts can raise circulating levels of β-hydroxybutyrate to 1.0 mM, but limitations exist with gastrointestinal tolerability and the high mineral load (33–35). Intravenous ketone salts can achieve higher and more sustained levels of circulating β-hydroxybutyrate levels (up to 3.5 mM) than orally delivered ketone salts (36). However, intravenous ketone salts are still limited by concerns about the associated mineral load (35, 37).

Ketone esters are another exogenous ketone substrate and are comprised of β-hydroxybutyrate esterified to precursor molecule. The ketone ester most prominent in published literature is in the form of (R)-3-hydroxybutyl (R)-3-hydroxybutyrate (38). It is highly tolerable and considered to be generally recognized as safe (GRAS) as a food ingredient by the FDA (38). After consumption, (R)-3-hydroxybutyl (R)-3-hydroxybutyrate is hydrolyzed to D-β-hydroxybutyrate and (R)-1,3-butanediol which is metabolized to D-β-hydroxybutyrate and acetoacetate in the liver (39). Oral ingestion can raise circulating β-hydroxybutyrate levels up to 5 mM within 30 minutes (39, 40). The most significant limitation of ketone esters is that they are expensive to produce and not available on a prescription basis. Currently, only two companies manufacture ketone esters, and they are primarily sold for sports performance. Other limitations of oral ketone ester ingestion include its rapid metabolism, necessitating multiple doses to maintain β-hydroxybutyrate levels, and its poor taste. These concerns are less likely to be problematic for patients receiving treatment in an intensive care setting (27). Another possible limitation of oral ketone ester administration is that ketone esters are only available for oral, nasogastric, and orogastric administration. Nasogastric and orogastric administration of ketone esters may not be possible in some patients as placement of the gastric tube may increase intracranial pressure (41). No studies to date have examined intravenous administration of ketone esters. A better understanding of how orally administered ketone salts and esters behave in the body is needed before they may be administered intravenously. See Table 1 for a summary of exogenous ketone compounds.

**Table 1:** A summary of exogenous ketone compounds.

**Exogenous ketone compound**	**Method of administration**	**Model(s)**	**Typical adult dosage**	**Plasma BHB concentration**	**Limitations**	**References**
Medium chain triglycerides (MCT)	Oral	Rodent Human	15–30 g of MCT	0.5 mM	Poor GI tolerability Low levels of ketosis Multiple doses required to maintain BHB level	(31, 32)
Ketone salt	Oral	Rodent Human	10–20 g of BHB	1 mM	Poor GI tolerability Low levels of ketosis Multiple doses required to maintain BHB level Associated mineral load	(33–35)
Ketone salt	Intravenous infusion	Pig Human	20 μmol/kg/min	Up to 3.5 mM	Associated mineral load	(36, 37)
Ketone ester	Oral	Rodent Human	573 mg /kg body mass	Up to 5 mM	Poor taste Multiple doses required to maintain BHB level	(27, 39, 40)

### Metabolic Efficiency of Ketones

Several mechanisms of ketone body metabolism may improve recovery under the pathological conditions of a TBI. First, the ketone body, β-hydroxybutyrate, can be metabolized into acetyl-CoA with four enzymes compared to the ten enzymes required for glucose to enter the tricarboxylic acid (TCA) cycle. Second, β-hydroxybutyrate bypasses the need to use the pyruvate dehydrogenase complex to enter the TCA cycle. In TBIs, the pyruvate dehydrogenase complex is inhibited (42). The pyruvate dehydrogenase complex may be a critical factor leading to long-term energy deficits as its inhibition hinders the production NADH and FADH_2_ in the TCA cycle, which disrupts the ability of the electron transport chain to generate ATP (42). Inhibition of the pyruvate dehydrogenase complex also decreases brain oxygen utilization and increases the ratio of lactate to pyruvate, both of which are indicative of impaired mitochondrial ATP production and decreased cellular phosphorylation energy (20). Because the metabolism of β-hydroxybutyrate does not utilize the pyruvate dehydrogenase complex, ketones may be advantageous to help restore deficits in cerebral energy.

Ketone bodies also consume less NAD+ than glucose to produce ATP. Both glucose and β-hydroxybutyrate pathways produce two molecules of acetyl-CoA, which subsequently enter the TCA cycle to generate ATP. Although glucose generates a higher final yield of ATP and reduces four molecules of NAD+, β-hydroxybutyrate reduces only one molecule of NAD+ during acetyl-CoA synthesis (43). The decreased use of NAD+ by ketones to form acetyl-CoA can increase the amount of NAD+ available to help maintain mitochondrial function, increase activation of antioxidant and pro-survival pathways, and decrease PARP-1-dependent cell death (44). By administering exogenous ketones, the metabolic efficiency of ketones can help spare cytosolic NAD+ pools, thereby increasing cell survival.

### Neuroprotective Effects of Ketones

In addition to being an efficient energy substrate for the injured brain, ketone bodies also have various neuroprotective effects. In TBIs, oxidative stress has been linked to many aspects of secondary injury mechanisms including lipid peroxidation, protein oxidation, DNA damage, electron transport chain inhibition, ionic dysregulation, and protease activation (7, 45). Ketone bodies have been shown to reduce oxidative stress by inhibiting the production of reactive oxygen species, preventing lipid peroxidation and protein oxidation, and increasing levels of antioxidant proteins (46). Because ketone bodies reduce oxidative stress, less NAD+ is depleted through enzymatic repair activity and more NAD+ is available for other cellular processes. The attenuation of oxidative stress by ketone bodies has a significant neuroprotective effect by improving cellular function and reducing energy deficits.

Ketone bodies have also been shown to regulate inflammatory responses. In TBIs, inflammatory responses can be both detrimental and beneficial. Inflammatory processes are crucial to recovery because these processes help clear debris and facilitate regeneration mechanisms. However, excessive inflammation can be harmful and contribute to the development of cerebral edema, a breakdown of the blood-brain barrier, and neurodegeneration (47, 48). In TBIs, the mechanistic target of rapamycin (mTOR) signaling pathway and NLRP3 (NOD-, LRR-, and pyrin domain-containing protein 3) inflammasome have been shown to be particularly relevant in respect to inflammation and neurodegeneration (49, 50). The mTOR signaling pathway regulates many aspects of cell growth, metabolism, proliferation and survival, and is upregulated in TBIs (51). Ketone bodies have been shown to reduce mTOR expression through increased AMP-activated protein kinase activity (52). Various selective mTOR inhibitors, including rapamycin, have been shown to decrease neuroinflammation, neuronal death, astrogliosis, and neurodegeneration following TBI (49, 53). Targeting mTOR expression through ketones may represent an effective strategy to reduce inflammation and neurodegeneration.

Another way ketone bodies can reduce inflammation and neurodegeneration is through the inhibition of the NLRP3 inflammasome (54). In TBIs, the activation of the NLRP3 inflammasome leads to the release of caspase-1, which mediates secretion of pro-inflammatory cytokines and triggers pyroptotic cell death (50, 55). Ketone bodies have been shown to inhibit NLRP3-mediated inflammatory responses and could help regulate excessive inflammation in TBI (54, 56). To effectively manage TBI inflammation, interventions need to limit acute proinflammatory responses to the level needed for beneficial recovery mechanisms and prevent the development of chronic inflammation. Because ketones have the ability to regulate inflammatory responses, ketones may be an effective treatment strategy to control inflammation.

Ketone bodies may also improve global and regional cerebral blood flow (52, 57). In TBIs, reductions in cerebral blood flow are seen in the acute phases and persist longer in patients with persistent TBI symptoms (58, 59). The decrease in cerebral blood flow is simultaneously accompanied by the increased demand on glycolysis and persists throughout the prolonged decrease in glucose metabolism (8). This results in a supply and demand mismatch with glucose not being brought to the brain, which contributes to the post-traumatic energy crisis (8). Ketones have been shown to improve cerebral blood flow through reduced mTOR expression and increased nitric oxide synthase protein expression (52). Because a decrease in cerebral blood flow inhibits the brain's ability to deliver oxygen and metabolites for proper brain function and recovery, ketone mediated improvements in cerebral blood flow have the potential to help to alleviate the mismatch in metabolic supply and demand.

Ketone bodies can also aid in closing the mitochondrial permeability transition pore (MPTP). The MPTP is a protein complex embedded in the inner and outer mitochondrial membrane that is linked to apoptotic and necrotic cell death. Following a TBI, the MPTP opens as a result of calcium overload and decrease in the ratio of ATP/ADP (14, 60). It has been established that the opening of the MPTP plays a pivotal role in the secondary injury cascade by destroying the integrity of the inner mitochondrial membrane (61, 62). This allows ions, cofactors, and water to enter mitochondria, leading to swelling, the eventual rupture of the outer mitochondrial membrane, and necrotic and apoptotic cell death (63). The metabolism of ketone bodies can help address cell death in three ways. First, when the MPTP opens, ions enter the mitochondrial matrix decreasing the free energy (ΔG) release of ATP hydrolysis (14). Because ketones can increase the ΔG of ATP hydrolysis, ketones can help address energy deficits and improve cellular function (14). Second, ketones can increase the threshold of calcium-induced MPTP opening by decreasing mitochondrial reactive oxygen species levels (64, 65). Third, ketones can minimize apoptotic cell death by reducing cytochrome c release (66). Given the importance of mitochondria in cellular function, targeting the MPTP with ketones can help restore mitochondrial function to limit cell death.

## Limitations and Unknowns

Although evidence suggests ketones may be beneficial in TBI treatment, several aspects of treatment need to be investigated before exogenous ketones can become a valid therapeutic approach. More research is needed to understand differences in ketone metabolism due to age and sex, the optimal timing of ketone administration, and how to translate results from experimental models to human patients. See Table 2 for a summary of the current state of knowledge regarding ketone therapy and TBI treatment.

**Table 2:** A summary of the current state of knowledge of ketone therapy in TBI treatment, including known effects, limitations, and unknowns.

**Known effects**	**Summary**
Metabolically efficient	Ketone body metabolism mechanisms are energetically favorable under the pathological conditions of a TBI and help reduce cerebral energy deficits.
Reduce oxidative stress	Ketone bodies reduce oxidative stress by inhibiting the production of reactive oxygen species, preventing lipid peroxidation and protein oxidation, and increasing levels of antioxidant proteins.
Regulate inflammation	Ketone bodies help regulate inflammatory processes that contribute to neurodegeneration and cerebral edema through the mTOR signaling pathway and NLRP3 inflammasome.
Improve blood flow	Ketone bodies improve cerebral blood flow through reduced mTOR expression and increased nitric oxide synthase expression.
Limit cell death	Ketone bodies can limit necrotic and apoptotic cell death by closing the MPTP protein complex and restoring mitochondrial function.
**Limitations and unknowns**	**Summary**
Age	Pediatric animal models show a faster and more sustained increase in plasma ketone levels compared to adults. Ketone bodies may not be able to address ongoing secondary injury processes in adults as effectively.
Sex	Aspects of mitochondrial function in human and animal studies are sex-dependent under normal physiological conditions. It is unknown how sex-dependent differences in TBI-induced mitochondrial dysfunction affect pathological responses.
	Baseline ketone body levels are higher and rise significantly more following the administration of exogenous ketones in female TBI animal models. Further research is needed to understand why ketone metabolism differs between sexes and how those differences impact TBI pathophysiology.
Timing and duration of ketone supplementation	Improvements in metabolic function following exogenous ketone administration may be dependent upon whether the patient is in a hypermetabolic or hypometabolic state.
	The effects of long-term ketosis in TBI recovery are unknown.
Experimental models	Animal models of TBI feature a homogenous type of injury with variables such as age, sex, genetic background, and injury parameters being well controlled. This may not accurately replicate the heterogenic nature of human TBI.
Ketone levels	The optimal level of cerebral ketones and the most effective method of ketone supplementation to achieve such levels are unknown.
Metabolic monitoring	Minimally invasive and accurate ways to monitor ketone metabolism in both intensive care and outpatient settings are needed.

### Age

Before exogenous ketones can be used in TBI treatment, differences in ketone metabolism across different ages must be considered. Studies with pediatric and adult rats fed a ketogenic diet following a TBI indicate that the efficiency of ketone metabolism is age dependent (67–69). Pediatric rats show a faster and more sustained increase in plasma ketone levels compared to adult rats (68). Pediatric rats also show improvements in motor and cognitive function and decreases in lesion volume and number of degenerating neurons while adult rats show no significant improvement (67, 68). The mechanisms underlying the observed differences between pediatric and adult TBI patients remains to be determined (70). However, it is hypothesized such differences may be due to the differences in monocarboxylate transporter expression between pediatric and adult TBI patients (16). Monocarboxylate transporters are membrane carrier proteins that transport ketone bodies across the plasma membrane, and they are more abundant in pediatric rats than in adult rats following a TBI (69, 71). Less monocarboxylate transporter expression may explain why the increase in plasma ketone concentration is delayed in adults, and therefore possibly not able to address ongoing secondary injury processes as efficiently.

Considering the rapid pathological progression following TBI, alternative approaches, such as incorporating a period of fasting to increase monocarboxylate transporter expression, could help resolve issues of age-dependent effects (72). However, a more comprehensive understanding of the metabolic changes associated with both pediatric and adult TBIs should be considered in order to develop ketone based pharmacological interventions. The current understanding of pediatric pathophysiology is limited due to the complexities of incomplete brain maturation and differences in cerebral metabolic requirements throughout development (70). Furthermore, our understanding of adult TBI is still improving (70). A more complete understanding of both pediatric and adult TBI pathophysiology is necessary to maximize the neuroprotective benefits of ketones.

### Sex

Our current understanding of TBI pathophysiology is also limited because it is currently uncertain whether TBI pathophysiology differs between males and females. Historically, in TBI studies, fewer females have been recruited to clinical trials, and male rodents have been the predominant experimental injury model (73). Because brain anatomy, cellular pathways, and drug pharmacokinetics can be affected by sex, our understanding of sex differences in TBI pathophysiology and recovery is lacking. The underrepresentation of females in both animal and human trials limits successful pharmacological developments and clinical care.

Recent studies suggest that neuropathological, physiological, social, and behavioral outcomes in TBIs are sex dependent (73). In a review examining 156 human TBI studies and 43 animal TBI studies, human studies report worse overall outcomes in females, whereas animal studies report better overall outcomes in females (73). While this difference may be due in part to injury severity, sample size, and the type of experimental injury model, several possible mechanisms might contribute to sex differences in TBI outcomes (73). These mechanisms include differences in how males and females acquire TBIs, how the brain responds at a cellular and molecular level to injury, and patterns of comorbidities and extracerebral injuries (73). At the cellular and molecular level, genetics and sex hormones have been shown to influence inflammation, edema, oxidative stress, excitotoxicity, and mitochondrial function (74–77). Because mitochondrial dysfunction and the resulting energy disruptions are central to TBI pathophysiology and ketones can help address energy disruptions, understanding sex differences in mitochondria are crucial. Multiple aspects of mitochondrial function in both human and animal studies are different between males and females under normal physiological conditions (73). These differences may be heightened during times of stress or injury (73). However, sex differences in TBI-induced mitochondrial dysfunction have only been explored to a limited extent. No studies to date have examined TBI-induced mitochondrial dysfunction in females to the same degree as TBI-induced mitochondrial dysfunction in males (78). Further research is required to understand how mitochondrial function may affect functional outcomes across both sexes in both humans and animals.

In respect to TBI and ketones, only one study to date has examined TBI sex differences in combination with ketone therapy (78). In this study, researchers found that baseline plasma concentrations of β-hydroxybutyrate were significantly lower in females compared to males (78). However, plasma concentrations of β-hydroxybutyrate increased by over 500% in females compared to a 100% increase in males following administration of β-hydroxybutyrate (78). This suggests the differences in male and female metabolism could potentially have negative effects on the brain as it is not yet understood how the brain differentiates between neuroprotective and neurotoxic levels of circulating ketones (79). Further research is needed to understand why ketone metabolism differs between the sexes and how those differences impact TBI pathophysiology.

### Timing

The timing and duration of exogenous ketone administration could also have significant consequences on TBI outcomes and warrants further investigation. Following a TBI, changes in cerebral energy metabolism occurs in two stages. The first stage is an acute period of hypermetabolism associated with excitotoxicity and increased glucose utilization (78). The second stage is a prolonged period of hypometabolism associated with the inhibition of glycolysis and mitochondrial dysfunction (78). Only one study to date has examined how the metabolic state during the time of ketone administration affects ketone metabolism in human TBI (78). In this study, researchers found that, in males, administering exogenous β-hydroxybutyrate in the hypometabolic state improved all measures of metabolic function, including mitochondrial function, reactive oxygen species production, and tricarboxylic acid cycle intermediate production (78). In contrast, β-hydroxybutyrate produced deleterious effects in females when administered during either the hypometabolic or hypermetabolic state (78). These results suggest that the neuroprotective properties of β-hydroxybutyrate depend upon both sex and metabolic state. However, the results of this study may be limited because metabolic functions were only analyzed at a single time point following induction of TBI. Additional research using rodent models has shown that other aspects of TBI pathophysiology, such as cytoskeletal damage and inflammatory patterns, peak at different times in males and females (76, 80). Because other aspects of TBI pathophysiology vary in timing between sexes, it is likely that variations in metabolic function across time also occur. Thus, future studies will need to examine both males at females at different times following TBI.

While research has shown that ketone metabolism can be beneficial during the acute phase of TBI, more research is needed to understand the effects of long-term ketosis in TBI recovery (35, 81). In healthy populations, studies have shown that long-term ketosis has no significant influence on long-term potentiation, synaptic function, and plasticity (82, 83). However, no studies to date have examined the long-term effects of ketosis specifically in TBI. It remains unclear whether long-term ketosis in TBI patients causes adverse effects. Future research will need to examine the optimal duration of exogenous ketone supplementation and any potential long-term effects.

Moderate-to-severe TBIs present an additional challenge in studying the timing of ketone administration due to the presence of additional complications. Although the secondary injury mechanisms of excitotoxicity, ionic disturbances, mitochondrial dysfunction, inflammation, and hypoglycemia still occur in moderate and severe TBIs, these issues are not the most pressing concern in early treatment. Early treatment of moderate-to-severe TBI primarily focuses on managing neurocritical care issues, such as multiple organ dysfunction syndrome, paroxysmal sympathetic hyperactivity, hemorrhagic progression, and coagulopathy (84). The administration of ketones in moderate-to-severe TBI with acute complications may not be beneficial and is largely unstudied in human TBI and animal models.

One barrier to studying the long-term effects of ketosis is that ketogenic diets are somewhat controversial, and high-quality clinical trials are lacking. Critics of the ketogenic diet, especially when it is employed primarily to lose weight, argue that the diet is a fad because conventional wisdom holds that high-fat diets are harmful, consumption of fiber and whole grains are essential, and ketogenic diets depend upon the consumption of animal products (85). Additionally, current research on ketogenic diets presents methodological challenges, such as attention to food quality, missing key micronutrients, and physical activity, that require studies to be interpreted with caution (85). However, evidence suggests that well-formulated ketogenic diets do not have any safety concerns for the general population (85). Although the ketogenic diet has a long history in clinical medicine and human evolution, high-quality clinical trials are needed to assess important questions about the long-term effects of ketogenic diets in healthy populations before the long-term effects of ketogenic diets can be studied in TBI.

### Experimental Models

To fully understand whether ketones are beneficial in treating TBIs, experimental models of TBI must be improved. Thus far, animal models of TBI have played an important role in characterizing TBI pathophysiology and in developing treatment strategies (15). However, animal models of TBI feature a homogenous type of injury with variables such as age, sex, genetic background, and injury parameters being well-controlled. In contrast, human TBI patients present a pathophysiological heterogeneity that can arise from the location, nature, and severity of the primary injury as well as preexisting factors and conditions such as age, health, sex, medication use, alcohol and drug use, and genetics (86). Furthermore, no studies to date have evaluated racial diversity in the pathophysiology of TBIs. As a result, no single animal TBI study can fully describe all possible pathophysiological consequences in human TBI. The heterogenic nature of TBIs may explain why the majority of pharmaceutical drugs identified to be effective in animal TBI models have failed in phase II and phase III clinical trials (87). The failure of translating preclinical animal studies into clinical settings underscores the need for new animal models of TBI to be developed and for existing models to be modified.

While early TBI research took place in rodent models and provided a fundamental understanding of TBI mechanisms, differences in rodent and human TBI pathophysiology exist. Because studying animal models with similar pathophysiological responses to humans is crucial to improving TBI treatments, the pig has become an increasingly prevalent TBI model (88). The pig is a more ideal model for TBI than rodents because its brain is closer in size, structure, composition, and development to the human brain and its immune system, inflammatory responses, and vasculature are more comparable to humans (88). Additionally, newly developed genetically engineered pigs with comorbidities and specific genetic factors associated with TBI may be helpful to test the effectiveness of novel therapeutic agents, including ketones, under conditions that increase the prevalence of TBI complications (88). Although the pig brain has numerous advantages that would help improve understanding human TBI, pig and human brains are not identical in pathological responses to injury (88). Direct comparisons of human and pig brain TBI pathophysiological responses are lacking. Future studies that examine pathological differences and similarities between both species are needed to progress in developing more effective TBI treatments.

Thus far, the majority of TBI and ketone research has used rodent models. No studies to date have examined ketone administration in TBI pig models. However, a study examining mitochondrial function in response to cyclosporine administration in a TBI pig model showed that intravenous cyclosporine administration improved brain metabolism and mitochondrial function by closing the mitochondrial permeability transition pore (60). These results offer promise that ketones could also improve brain metabolism and mitochondrial function by providing an energy source to close the mitochondrial permeability transition pore in a large TBI model similar to humans.

The majority of TBI research in animals has used a controlled cortical impact (CCI) model, shock tube model, lateral fluid percussion injury (FPI) model, or weight drop model to replicate the mechanisms of TBI pathophysiology. The CCI model best replicates focal injuries with localized tissue deformation resulting from a blow to the head, such as in sports-related TBI or violent assaults (89, 90). The shock tube model best replicates diffuse injuries caused by acceleration and deceleration forces, such as in car accidents and blast-related military TBI (89). The FPI model replicates TBI with both focal and diffuse injury patterns, and the weight drop model can replicate focal or diffuse injuries depending on the technique used (89). Because each of these TBI models have different associated injury mechanisms and pathological consequences, utilizing multiple models in ketone and TBI research would allow the effectiveness of ketones in TBI treatment to be evaluated across various types of civilian and military TBI.

### Human Traumatic Brain Injuries and Ketones

Most evidence regarding the benefits of ketones in treating TBI has come from animal studies. Ketone therapy research in human TBI is only beginning to look at the safety and efficacy of ketone supplementation. The most recent research includes a phase II clinical trial that found an enterally administered ketogenic formulation can be safely administered to TBI patients while improving glucose control and an ongoing phase I clinical trial investigating the safety, feasibility, and effectiveness of an enteral ketogenic formulation for severe TBI (91, 92). Another recent study examined the ketogenic diet and medium chain triglyceride supplementation in post-concussion syndrome and reported improvements in visual memory and post-concussion syndrome symptomology (93). Together, these studies have established a physiological and symptomatic rationale for future clinical studies to examine ketone therapy in TBI.

Although not yet widely studied in human TBIs, ketones have been used more frequently in other health conditions with similar pathophysiological consequences. In type 2 diabetes, where cognitive impairment is associated with cerebral glucose hypometabolism, intravenous administration of ketone salts improves working memory performance (94). In the process of aging, where age-based cognitive impairment has been linked with cerebral glucose hypometabolism, ketosis achieved through both the ketogenic diet and ketone ester supplementation improves functional communication between brain regions (95). In Alzheimer's, where disease progression is linked to impaired glucose metabolism and neuronal cell death, the ketogenic diet mitigates neurodegeneration processes (96–98). In Parkinson's disease, where cells experience abnormal energy metabolism, oxidative stress, inflammation, and increased apoptosis, the ketogenic diet improves both motor and non-motor symptoms (99, 100). In migraines, where migraines are thought to be triggered by cerebral energy deficiencies or oxidative stress, the ketogenic diet reduces migraine frequency, severity, and medication use (101, 102). In epilepsy, where mitochondrial bioenergetics are impaired, the ketogenic diet has been used since the 1920s in children to reduce seizures and has been increasingly used in adults with drug resistant epilepsy (103, 104). Several clinical trials support the belief that ketogenic diets reduce seizures in both children and adults (105, 106). See Table 3 for a summary of how ketones have been used in health conditions relating to TBI pathophysiology.

**Table 3:** A summary of how ketones have been used in health conditions relating to TBI pathophysiology.

**Health condition**	**Study**	**Ketogenic agent**	**Findings**
Type 2 diabetes	(94)	Ketone salt	Improved working memory performance
Aging	(95)	Ketone ester	Improved brain network stability
Alzheimer's	(96)	Ketogenic diet	Improved metabolism, reduced neuronal cell death
Parkinson's	(99)	Ketogenic diet	Improved motor and non-motor symptoms
Migraines	(101)	Ketogenic diet	Reduction in migraine frequency, severity, and medication use
Epilepsy	(105)	Ketogenic diet	Reduction in seizure frequency in children
	(106)	Ketogenic diet	Reduction in seizure frequency in adults

The studies examining ketones in the treatment of various health conditions involving disruptions in cerebral metabolism focus on outcomes and symptoms that differ from TBI treatment measures. However, the underlying mechanisms behind why ketones help improve such outcomes provides support for why and how ketones can address similar mechanisms present in TBIs. Support for use of the ketogenic diet and forms of exogenous ketones in other health conditions involving disruptions in cerebral metabolism offer promise for future TBI and ketone studies in humans.

### Metabolic Monitoring

For ketones to be studied more extensively in clinical settings, methods of monitoring cerebral metabolism must be improved. Positron emission tomography (PET) is a common, non-invasive imaging approach used for various neurological problems, including TBI (107). In PET, ^18^F is attached to deoxyglucose and can quantify the proportion of glucose uptake and phosphorylation, and ^11^C is attached to acetoacetate to quantify the proportion of ketone body uptake (78, 107, 108). While PET imaging is informative, it has several limitations, including limited spatial resolution, the amount of radioisotope that can be delivered to injured tissue relative to normal tissue, procedural aspects that may not be feasible for every patient, and the inability to continuously monitor cerebral metabolism (78, 107). The most significant limitation of PET is that the absolute quantification of cerebral glucose and ketone uptake requires a complex and invasive procedure not suitable for clinical routine. The absolute quantification of metabolites requires an arterial input function that describes a non-metabolized compound's concentration as a function of time (109). The determination of an arterial input function is an invasive procedure and can disturb a patient's physiological status (109). While standardized uptake values can be used to provide semi-quantitative measures of cerebral metabolite uptake, standardized uptake values lack an absolute physiological scale that limits the ability to accurately assess potential TBI treatments (109, 110).

An image-derived input function is a non-invasive alternative to an arterial input function that can accurately quantify absolute physiological values. The most accurate method of calculating an image-derived input function uses a fully integrated positron emission tomography-magnetic resonance imaging (PET-MRI) system (111). Until recently, PET and MRI could not be performed simultaneously because the magnets in MRI scanners interfered with detectors in PET scanners, and merging separate PET and MRI images presented logistical challenges that made calculating an accurate image-derived input function a challenge (112). Recent advances in research have developed PET-MRI systems that can be used in combination with computational frameworks to generate an image-derived input function that accurately reflects arterial input function values (109, 111). Because the integrated PET-MRI system is a relatively new technology, it is primarily used in research settings and not yet available for routine clinical use. The first integrated PET-MRI systems in the United States were installed in 2010, and in 2017, there were 30 PET-MRI systems in the United States (113). As PET-MRI systems become more prevalent, the absolute quantification of physiological metabolite levels through image-derived input functions will allow more accurate evaluations of potential TBI treatments, including ketones.

Cerebral microdialysis (CMD) is an alternative approach to monitor cerebral metabolism. CMD allows semi-continuous bedside monitoring of cerebral metabolism by sampling parenchymal extracellular fluid with a probe to obtain concentrations of specific biomarkers (114). Various metabolites involved in cerebral glucose, ketone, and oxidative metabolism can be monitored *via* CMD (18, 29, 81). Advances in CMD use and interpretation have allowed CMD to become a routine neuromonitoring tool to help guide individualized treatment in neurointensive care TBI patients (114, 115). However, CMD is limited because metabolites can only be measured in a very narrow zone surrounding the CMD probe (116). While this narrow measurement zone can help understand the metabolism of specific areas in the brain, CMD does not provide a complete metabolic picture of the entire brain. Because cerebral energy metabolism is usually very different in different parts of the brain in TBI patients, treatments targeting specific aspects of cerebral metabolism based on CMD measurements may not prove effective for the entire brain (116). Another limitation of CMD is that CMD is generally only used in intensive care units with severe TBI patients and is not suitable for milder forms of TBI, such as concussions. While CMD has been used in animal studies to study milder forms of TBI, its practicality in clinical settings is limited.

Options for monitoring ketone metabolism in milder forms of TBI and outpatient settings are limited because cerebral ketone concentrations cannot be determined with the methods currently available. These options include urinalysis, breath analyzers, and blood meters. Urinalyses methods predominately use a urine ketone stick test, which can give a semi-quantitative and inexpensive measure of acetoacetate (117). However, the quantity of ketones in urine does not equate to plasma ketone concentrations because urine tests do not detect β-hydroxybutyrate, which is the predominate circulating ketone (117). Breath analyzers are a convenient and fast way to measure ketone levels. The main advantage of ketone breath analyzers is that they allow an unlimited number of ketone measurements to be easily conducted after the user purchases a breath analyzer device. However, ketone breath analyzers only measure the ketone body acetate, which is exhaled as a byproduct of fat metabolism. While acetate can be a reliable marker of endogenous ketosis and fat metabolism, levels of exogenous ketones may not be accurately detected (118). Furthermore, only one ketone breath analyzer (Biosense) on the market has been shown to have a high level of accuracy (119). Ketone blood meters measure capillary concentrations of β-hydroxybutyrate and are an accurate way of measuring circulating levels of ketones (117). The biggest limitation of ketone blood meters is that ketone test strips are relatively expensive, and finger prick tests may need to be performed multiple times throughout the day (117). One way to reduce the costs and improve the convivence of accurately measuring ketone levels is through developing a continuous ketone monitor, similar to that of continuous glucose monitoring systems. The first proof of concept of a continuous ketone monitor that can be placed within the subcutaneous tissue to measure ketone concentration for 14 days was recently completed (120). However, more research is needed to evaluate the performance of the device in various patient populations (120). Thus, to improve TBI treatment strategies in both intensive care and outpatient settings, it will be essential to establish technologies that can monitor ketone metabolism in accurate and minimally invasive ways. Improved metabolic monitoring technology will allow the development of quantitative measures to guide the ketone therapy methodology and assess the effectiveness of ketone therapy in TBI.

### Ketone Levels

To translate experimental data supporting the benefits of ketone metabolism in TBI into clinical practice, the optimal level of cerebral ketone bodies must be determined. Thus far, most ketone research has focused on plasma levels of ketone bodies, and limited data exists about ketone body levels in the brain. A study exploring cerebral ketone metabolism following TBI in humans indicates that plasma ketone bodies are the primary source of cerebral ketone bodies (81). However, the level of cerebral ketone bodies required to effectively address TBI mechanisms and the level of plasma ketone bodies required to achieve such cerebral ketone body levels are unknown (91).

Achieving optimal cerebral ketone body levels is complicated by ketone body transport into the brain that requires monocarboxylate transports to cross the blood brain barrier. Even if plasma ketone body levels are high enough to theoretically reach optimal cerebral ketone body levels, ketone transport into the brain is limited by monocarboxylate transporters. Further complicating this issue is that monocarboxylate transporters are expressed significantly more in pediatric TBIs compared to adult TBIs (121). Therefore, strategies such as fasting may be needed to increase ketone transport across the blood brain barrier to provide sufficient levels of cerebral ketone bodies in adult patients.

In addition to understanding the level of plasma and cerebral ketones needed to target TBI mechanisms, the optimal method of increasing cerebral ketone body levels must be determined. A ketogenic diet enriched with medium chain triglycerides provides only small increases in plasma ketone body levels and may not be sufficient to increase cerebral ketone body levels (81, 91). Other methods, such as intermittent feeding and exogenous ketone salts and esters, may be needed to raise ketone body concentrations high enough to serve as a cerebral metabolic substrate. Intermittent rather than continuous methods of feeding may be helpful to promote the metabolic switch from reliance on glucose to reliance upon ketone bodies and promote increased ketone body uptake by monocarboxylate transporters (122). Exogenous ketone delivery through ketone esters and ketone salts could be more effective in raising ketone body levels instead of using a medium chain triglyceride enriched diets to produce endogenous ketone bodies (81). Ketone esters may be the best approach to raise plasma ketone body levels to therapeutic concentrations as ketone esters result in a higher plasma ketone body level increase. However, no studies to date have directly compared the effects of different ketogenic agents in TBIs. Future studies in both human and animal models are needed to determine the optimal level of plasma and cerebral ketone body levels and the most effective method to achieve such levels.

## Discussion

Our understanding of TBI pathophysiology has drastically improved in the past 20 years. However, more than 30 large clinical trials have failed to identify targeted pharmacological therapies in TBI patients that improve recovery outcomes. The heterogenic nature of TBI presents challenges in both TBI research and treatment. Because disruptions in cerebral metabolism are a commonality across all forms of TBI, ketone therapy may be a valid therapeutic approach to improve recovery by boosting cerebral energy metabolism, mitigating secondary injury mechanisms, and exerting neuroprotective effects against acute and progressive neurodegenerative processes. The mechanisms of ketone therapy for TBI are beginning to be understood, however more research is needed in both animal models and humans to elucidate how to effectively utilize ketones and translate pre-clinical findings into clinical practice. Specifically, age-dependent differences in ketone metabolism, sex differences in the response to ketone administration, the timing and duration of ketone administration, and optimal level of cerebral ketone levels needs to be explored. Advances could also be made to improve TBI modeling techniques and methods of monitoring cerebral metabolism. Overall, ketone therapy holds significant promise as an option for TBI treatment, and preliminary evidence strongly warrants further investigation of ketones in TBI treatment.

## Author Contributions

SD confirms sole responsibility for the conception and design and manuscript preparation of this review.

## Conflict of Interest

The author declares that the research was conducted in the absence of any commercial or financial relationships that could be construed as a potential conflict of interest.

## Publisher's Note

All claims expressed in this article are solely those of the authors and do not necessarily represent those of their affiliated organizations, or those of the publisher, the editors and the reviewers. Any product that may be evaluated in this article, or claim that may be made by its manufacturer, is not guaranteed or endorsed by the publisher.
